# Investigation of M2 macrophage-related gene affecting patients prognosis and drug sensitivity in non-small cell lung cancer: Evidence from bioinformatic and experiments

**DOI:** 10.3389/fonc.2022.1096449

**Published:** 2022-12-15

**Authors:** Zhen Zeng, Jiachen Yu, Zhuo Yang, Kangming Du, Yuewei Chen, Lei Zhou

**Affiliations:** ^1^ Department of Emergency, Hospital of Chengdu University of Traditional Chinese Medicine, Chengdu, Sichuan, China; ^2^ Peking Union Medical College, Chinese Academy of Medical Sciences, Beijing, China; ^3^ Department of Cardiology, Hospital of Chengdu University of Traditional Chinese Medicine, Chengdu, Sichuan, China; ^4^ Department of Cardiothoracic Surgery, Hospital of Chengdu University of Traditional Chinese Medicine, Chengdu, Sichuan, China; ^5^ Pain Management, Hospital of Chengdu University of Traditional Chinese Medicine, Chengdu, Sichuan, China

**Keywords:** M2 macrophages, HNMT, model, immunotherapy, drug

## Abstract

**Background:**

The progression process of lung cancer can be accelerated by M2 macrophages. However, genes that affect M2 macrophage polarization remain unidentified.

**Methods:**

The Cancer Genome Atlas, Gene Expression Omnibus, and Arrayexpress databases were used to obtain open-access data. The analysis of public data was mostly performed with R studio. The RNA levels of specific genes were detected using quantitative real-time PCR. The proliferation ability of the cells was assessed by CCK8, colony formation, and EdU assays.

**Results:**

Based on the multiple datasets, we noticed a poor prognosis in patients with high M2 macrophage infiltration. There were 114 genes differentially expressed between high and low M2 macrophages infiltrated samples, regarded as M2 macrophage-related genes. Subsequently, a prognosis prediction signature consisting of ABHD5, HS3ST2, TM6SF1, CAPZA2, LEPROT, HNMT, and MRO was identified and presented a satisfactory performance. The pathway enrichment results revealed a positive correlation between riskscore and enrichment scores for most immunotherapy-related positive terms. Also, there might be an increase in genomic instability among patients at high risk. Interestingly, low risk patients are most likely to benefit from PD-1 therapy, while high risk patients may benefit from CTLA-4 therapy. Meanwhile, the estimated IC50 of seven drugs differs significantly between two risk groups, including Cisplatin, Docetaxel, Doxorubicin, Gefitinib, Paclitaxel, Sunitinib and Vinorelbine. Moreover, further experiments indicated that HNMT was overexpressed and can enhance the proliferation ability in lung cancer cells.

**Conclusions:**

In summary, our study identified the molecules significantly affecting M2 macrophage infiltration and identified a prognosis signature that robustly indicated patients prognosis. Moreover, we validated the cancer-promoting effect of HNMT using *in vitro* experiments.

## Introduction

The world over, lung cancer is responsible for a disproportionate number of cancer related-deaths (1). Among the pathological types of lung cancer, non-small cell lung cancer (NSCLC) is the most prevalent, which consists of lung squamous cell carcinomas (LUSC) and lung adenocarcinomas (LUAD) (2). Lung cancer prevalence is often multifactorial, which brings difficulties to its prevention and treatment (3). For lung cancer patients with early-stage, surgical resection combined with chemoradiation may provide a better prognosis than chemotherapy alone, but their efficacy is still limited for the metastatic stage (4). Consequently, identifying effective molecular targets for diseases is imperative (5).

As research progresses, it is gradually understood that tumor occurrence, growth, and development are strongly influenced by the tumor microenvironment (6). Research has shown that tumor-infiltrating immune cells make up the majority of the microenvironment (7). Among these, macrophages might play a non-negligible role. Macrophages inside tumors have been defined as tumor-associated macrophages (TAMs). Generally, M1 macrophages hamper tumor development, whereas M2 macrophages contribute to tumor progression (8). Recently, more and more research is being devoted to understanding how TAMs work in specific tumors. In lung cancer, Xu and their colleagues demonstrated that the growth of lung cancer and metastasis can be inhibited by astragaloside IV through its modulation of macrophage M2 polarization through AMPK signaling (9). Wu and their colleagues showed that the succinate derived from cancer cells could contribute to macrophage polarization, further enhancing tumor metastasis through the succinate receptor (10). In the tumor microenvironment, macrophage M2 polarization was influenced by multiple factors. Meanwhile, the abnormal expression of specific genes could affect local tissue recruitment of TAMs, especially M2 macrophages. Exploration of the factors associated with macrophage M2 polarization could help us get an improved understanding of tumor progression and metastasis, allowing the identification of new targets for clinical therapy.

Bioinformatics can enhance people’s understanding of diseases (11, 12). In our study, infiltration of M2 macrophages was observed as a cancer-promoting effect of NSCLC in several independent cohorts. Meanwhile, Next, we established a prognosis prediction signature based on seven M2 macrophage-related genes ABHD5, HS3ST2, TM6SF1, CAPZA2, LEPROT, HNMT, and MRO, which showed great prediction efficiency. Further, the potential difference in different risk groups was investigated, including pathway enrichment, and genomic mutation exploration. Interestingly, low risk patients are most likely to benefit from PD-1 therapy, while high risk patients may benefit from CTLA-4 therapy. Also, the estimated IC50 of seven drugs differs significantly between two risk groups, including Cisplatin, Docetaxel, Doxorubicin, Gefitinib, Paclitaxel, Sunitinib and Vinorelbine. Moreover, further experiments indicated that HNMT was overexpressed and can enhance the proliferation ability in lung cancer cells. Our study can improve the understanding of researchers on M2 macrophages in lung cancer.

## Methods

### Open-accessed data acquisition

NSCLC datasets with complete expression matrix data and clinical annotations have been searched exhaustively in public databases. Finally, six independent NSCLC cohorts were identified in our analysis, including TCGA-LUAD, TCGA-LUSC, E-GEOD-30219, GSE37745, GSE50081 and GSE68465. The expression profile was transcripts per kilo-base million (TPM) type. Using the reference file Homo_sapiens.GRCh38, probe annotation was conducted. GSE68465 (GPL96), GSE50081 (GPL570) (13), and GSE37745 (GPL570) were identified from the GSE database (14). E-GEOD-30219 (GPL570) was identified from the Arrayexpress database. Considering the same platforms of GSE50081, GSE37745, and E-GEOD-30219, the intra-batch and inter-batch effects of these were corrected using the sva package. A standardization procedure was followed before data analysis (15).

### Prognosis model establishment and validation

Following the identification of M2 macrophage-related DEGs, we screened for prognosis-related genes sequentially using univariate Cox analysis, LASSO regression, and multivariate Cox analysis. Finally, the prognosis model was established with the following formula: Riskscore = Σcoef*Exp(genes).

### Nomogram plot, calibration curve and decision curve

Clinical features and riskscore of patients were combined to establish a nomogram. Meanwhile, the evaluation of the accuracy of the nomogram was conducted using the calibration curve and decision curve analysis (DCA).

### Immune infiltration quantification and pathway enrichment

Based on a CIBERSORT algorithm, an evaluation of the microenvironment surrounding NSCLC tumors revealed 22 types of infiltrating immune cells (16). Biological investigation in different groups was conducted using the Gene Set enrichment analysis (GSEA) algorithms based on Hallmark, Gene Ontology (GO) and Kyoto Encyclopedia of Genes and Genomes (KEGG) gene sets (17). Single sample gene set enrichment analysis (ssGSEA) was used to calculate the correlation between riskscore and specific pathway score (18).

### Genomic instability analysis

Tumor mutation burden (TMB) measures how many base mutations are found in a 1Mb region of DNA. TMB was calculated and compared in different groups based on the data from TCGA. Using previously sorted data, the microsatellite instability (MSI) of NSCLC patients was assessed (19). The R package maftools were utilized to identify the mutated genes in different groups with statistically significant (20).

### Sensitivity analysis of immunotherapy and chemotherapy

The sensibility of immunotherapy was quantified with the Tumor Immune Dysfunction and Exclusion (TIDE) algorithm. Also, the Subclass mapping algorithm was used to assess the genomic similarity between different risk patients and 47 immunotherapy-responding patients (21). Drug sensitivity analysis was performed using the Genomics of Drug Sensitivity in Cancer (GDSC) database (22).

### Cell culture and quantitative real-time PCR

The BEAS-2B, H838, A549, H441 and H1299 were routine storage in the laboratory and cultured under standard conditions. A total RNA extraction kit was applied for RNA extraction. Processes of qRT-PCR were completed using the standard procedures. Primers used were as follows: HNMT, forward, 5’-GTTTGCTTGGCATAAGGAGACA-3’, reverse, 5’-TGATCCGTACTTTTTCCACAGC-3’, GAPDH, forward, 5’-GCAAATTCCATGGCACCGT-3’, reverse, 5’-TCGCCCCACTTGATTTTGG-3’.

### Cell proliferation assay

Evaluation of cell proliferation ability was conducted using the CCK8, colony formation and EdU assay according to the standard procedures (23).

### Statistical analysis

All the statistical analysis was conducted using R software (version 4.0.0), SPSS (version 23.0) and GraphPad Prism 8. Briefly, the significance of the difference is determined by the p-value < 0.05. For continuous variables with normal distribution, the Student T test is used. Data that were not normally distributed were compared using Mann-Whitney U tests.

## Results

### Quantification of TAMs in NSCLC

**Figure 1:**
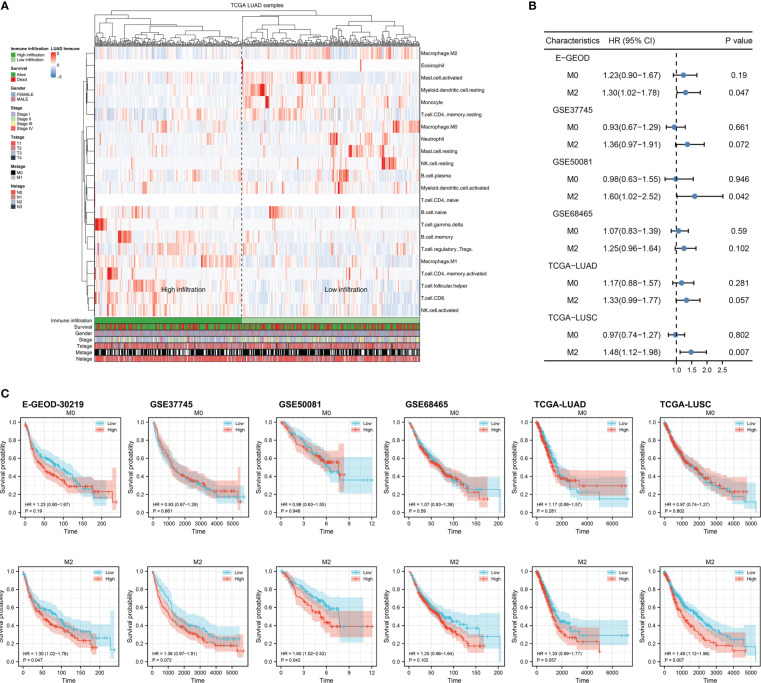
Exploration of M2 macrophage in lung cancer **(A)** The infiltration level of M2 macrophage was quantified using the CIBERSORT algorithm; **(B)** The prognosis correlation of M0 and M2 macrophage in multiple independent lung cancer cohorts; **(C)** KM survival curves of M0 and M2 macrophage in multiple independent lung cancer cohorts.

Firstly, we quantified the immune microenvironment of the NSCLC tissue microenvironment, including TAMs (**Figure 1A**). Univariate Cox regression analysis indicated that in multiple independent NSCLC cohorts, M2 macrophages might exert a risk factor of patient OS, but M0 macrophages not (**Figure 1B**; M2 macrophages, E-GEOD, HR = 1.30, 95% Cl = 1.02-1.78; GSE37745, HR = 1.36, 95% Cl = 0.97-1.91; GSE50081, HR = 1.60, 95% Cl = 1.02-2.52; GSE68465, HR = 1.25, 95% Cl = 0.96-1.64; TCGA-LUAD, HR = 1.33, 95% Cl = 0.99-1.77; TCGA-LUSC, HR = 1.48, 95% Cl = 1.12-1.98, M0 macrophages, E-GEOD, HR = 1.23, 95% Cl = 0.90-1.67; GSE37745, HR = 0.93, 95% Cl = 0.67-1.29; GSE50081, HR = 0.98, 95% Cl = 0.63-1.55; GSE68465, HR = 1.07, 95% Cl = 0.83-1.39; TCGA-LUAD, HR = 1.17, 95% Cl = 0.88-1.57; TCGA-LUSC, HR = 0.97, 95% Cl = 0.74-1.27). The same trends of Kaplan-Meier (KM) survival curves was shown in **Figure 1C**.

### Biological pathway effect of M2 macrophages in NSCLC

**Figure 2:**
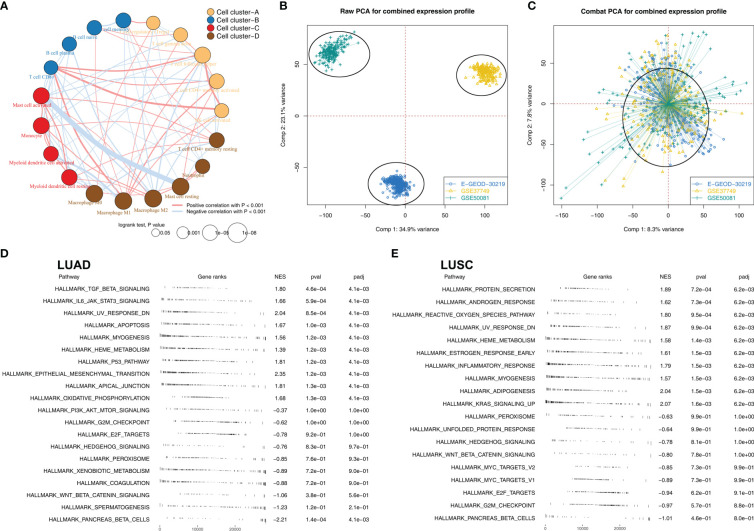
The biological role of M2 macrophages in lung cancer **(A)** Correlation analysis of the quantified 22 immune cells; **(B)** Significant batch effect was observed in E-GEOD-30219, GSE37749 and GSE50081; **(C)** Sva package was used for data combination; **(D)** GSEA analysis was performed to explore the biological pathway differences between high and low M2 macrophages LUAD samples; **(E)** GSEA analysis was performed to explore the biological pathway differences between high and low M2 macrophages LUSC samples.

**Figure 2A** illustrated that M2 macrophages were positively correlated with monocyte and activated mast, but negatively correlated with Tregs, plasma B cells, CD8+ T cells, memory B cells, activated NK cells and follicular helper T cells. We then tried to combine the E-GEOD-30219, GSE37749, and GSE50081 into a large population cohort for the same platform. Batch differences between these cohorts were significant (**Figure 2B**). Using sva package, the batch effect of these three NSCLC cohorts was remarkably decreased (**Figure 2C**). The GSEA analysis revealed that in LUAD patients with high M2 macrophages infiltration, pathways of TGF-β signaling, apoptosis, P53 signaling, the epithelial-mesenchymal transition were significantly activated, yet the PI3K/AKT/mTOR signaling, G2M checkpoint, E2F target was downregulated (**Figure 2D**). In LUSC patients with high M2 macrophage levels, pathways of protein secretion, androgen response, and reactive oxygen species were significantly upregulated, yet the peroxisome, unfolded protein response, and hedgehog signaling was downregulated (**Figure 2E**). For GO analysis, in LUAD patients with high M2 macrophage infiltration, the terms of white fat cell differentiation, abnormal cardiac exercise stress test and sialic acid binding were activated (**Figure S1A**); in LUSC patients with M2 macrophage infiltration, the terms of neurotransmitter gated ion channel clustering, regulation of systemic arterial blood pressure by circulatory renin angiotensin and sialic acid binding were activated (**Figure S1B**). For KEGG analysis, in LUAD patients with high M2 macrophage infiltration, the terms of melanoma, renal cell carcinoma and leishmania infection were activated (**Figure S1A**); in LUSC patients with high M2 macrophage infiltration, the terms of leishmania infection, lysosome and cell adhesion molecules cams (**Figure S2B**).

### Identification of M2 macrophages-related genes associated with patients prognosis

**Figure 3:**
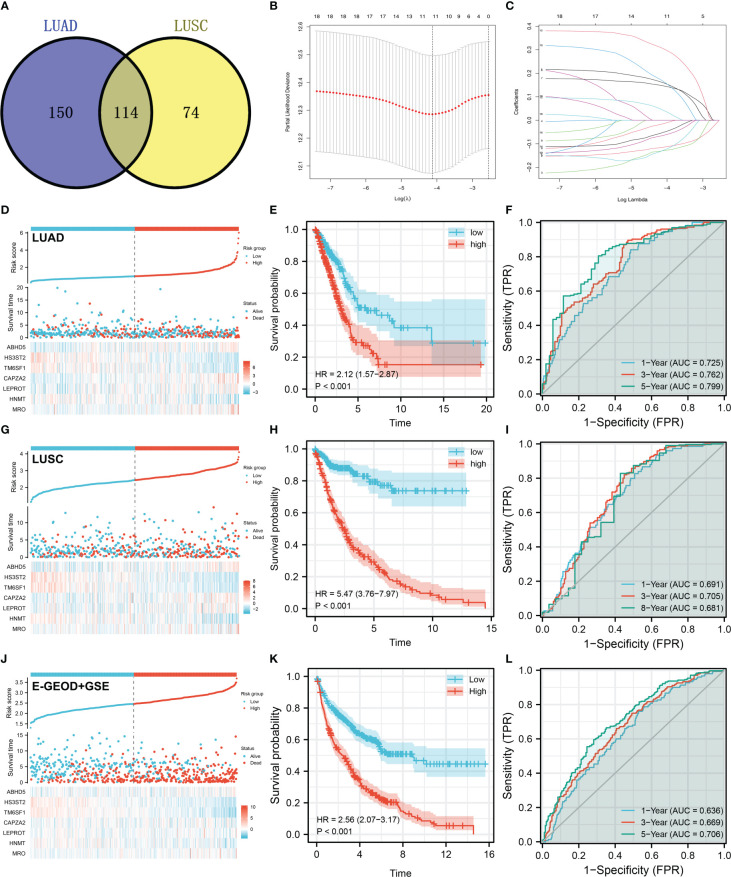
Prognosis model construction based on the M2 macrophage-related genes **(A)** A total of 114 genes were identified as M2 macrophage-related genes with the intersection of LUAD and LUSC data; **(B, C)** LASSO regression analysis was used for dimensionality reduction; **(D)** The overview of our prognosis model in the LUAD cohort; **(E)** KM survival curve was performed to explore the prognosis differences between high and low risk patients in LUAD cohort; **(F)** ROC curve was performed to evaluate the prediction efficiency of our model in LUAD cohort; **(G–I)** Model validation in LUSC cohort; **(J–L)** Model validation in E-GEOD + GSE cohort.

There were 114 genes differentially expressed between high and low M2 macrophages infiltrated samples, regarded as M2 macrophage-related genes (**Figure 3A**). Next, we aimed to identify a prognosis signature based on the M2 macrophage-related gene to robustly predict the patients OS. The TCGA-LUAD cohort was selected as the training cohort, and TCGA-LUSC and combined cohort (E-GEOD-30219 + GSE37749 + GSE50081) were used for validation. Univariate Cox regression analysis was firstly conducted to identify the molecules associated with patients OS with P < 0.05. A dimensionality reduction analysis was then conducted using the Lasso regression, and cross-validation was conducted 10 times (**Figures 3B, C**). Furthermore, multivariate Cox regression analysis indicated that ABHD5, HS3ST2, TM6SF1, CAPZA2, LEPROT, HNMT, and MRO were prominently associated with the risk of patients survival. These seven genes have been used to predict the survival rate of NSCLC patients with “riskscore = ABHD5 * 0.2406 + HS3ST2 * -0.1412 + TM6SF1 * -0.3109 + CAPZA2 * 0.1895 + LEPROT * 0.2919 + HNMT * -0.1554 + MRO * 0.3260”. Training cohorts with high risk were observed to have a higher proportion of dead cases (**Figure 3D**). KM survival curves revealed that patients with higher riskscores might have a worse outcome (**Figure 3E**). According to the ROC curve, our model was highly effective at predicting patients outcomes (**Figure 3F**, AUC of 1-, 3- and 5-year were 0.725, 0.762 and 0.799). In the TCGA-LUSC cohort, the prognosis prediction efficacy is still good (**Figures 3H, I**, HR = 5.47, P < 0.001; AUC of 1-, 3- and 5-year were 0.691, 0.705 and 0.681). The same conclusion was also found in the E-GEOD + GSE cohort (E-GEOD-30219 + GSE37749 + GSE50081) (**Figures 3J–L**, HR = 2.56, P < 0.01; AUC of 1-, 3- and 5-year were 0.636, 0.669 and 0.706).

### Development of a prognostic nomogram

**Figure 4:**
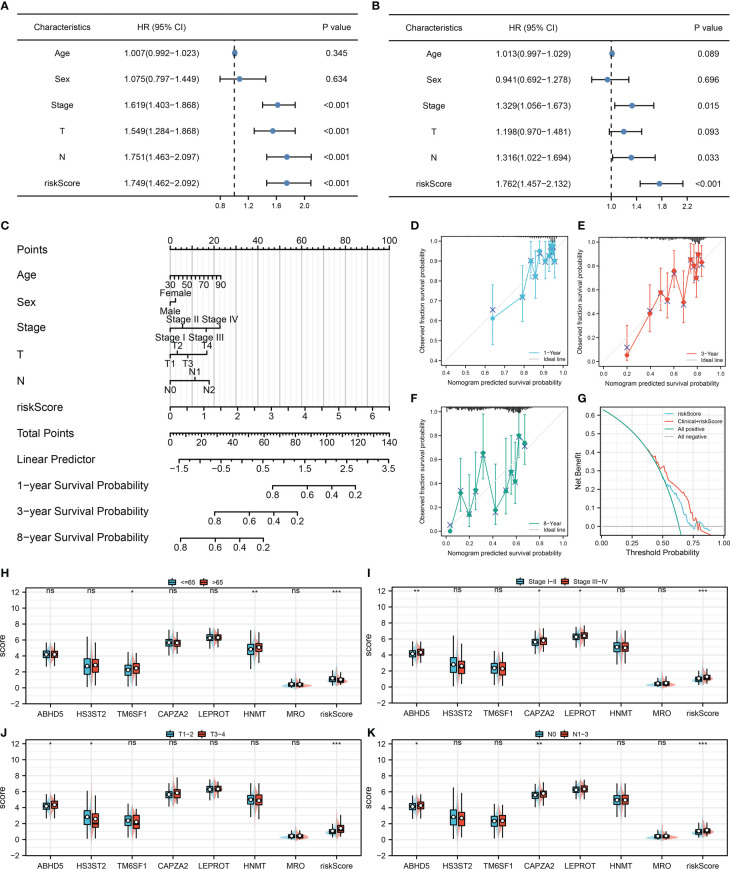
Nomogram and clinical correlation **(A, B)** Univariate and multivariate Cox regression analysis were performed to evaluate the independence of prognostic models; **(C)** Nomogram was constructed by combining the riskscore and clinical features; **(D–F)** Calibration curves of the nomogram; **(G)** DCA curve of the nomogram; **(H–K)** Clinical correlation of the model genes and riskscore.

Cox regression analysis was performed to further determine if our model could be a prognosis factor independent of traditional clinical features. Results of univariate Cox regression analysis demonstrated that some clinical features and riskscore were distinctively linked with patients survival (**Figure 4A**). Nevertheless, only riskscore remained an independent prognostic factor following multivariate Cox regression analysis (**Figure 4B**). Moreover, a nomogram was established that included five clinical variables and riskscore (**Figure 4C**). Based on the calibration plot, the data indicated good agreement between the real survival observation and the prediction for 1-, 3-, and 8 years (**Figures 4D–F**), and DCA analysis showed that the model with clinical features and riskscore had the best benefit to a treatment decision (**Figure 4G**). In addition, we assessed the significance of riskscore and seven model genes on correlations with clinicopathological parameters (**Figures 4H–K**). The result showed that ABHD5, CAPZA2, LEPROT and riskscore might be associated with worse clinical stage; ABHD5 and riskscore might be associated with worse T-classification, yet HS3ST2 was contrary; ABHD5, CAPZA2, LEPROT and riskscore might be associated with more progressive N-classification.

### Comparative genomic analyses of the model

**Figure 5:**
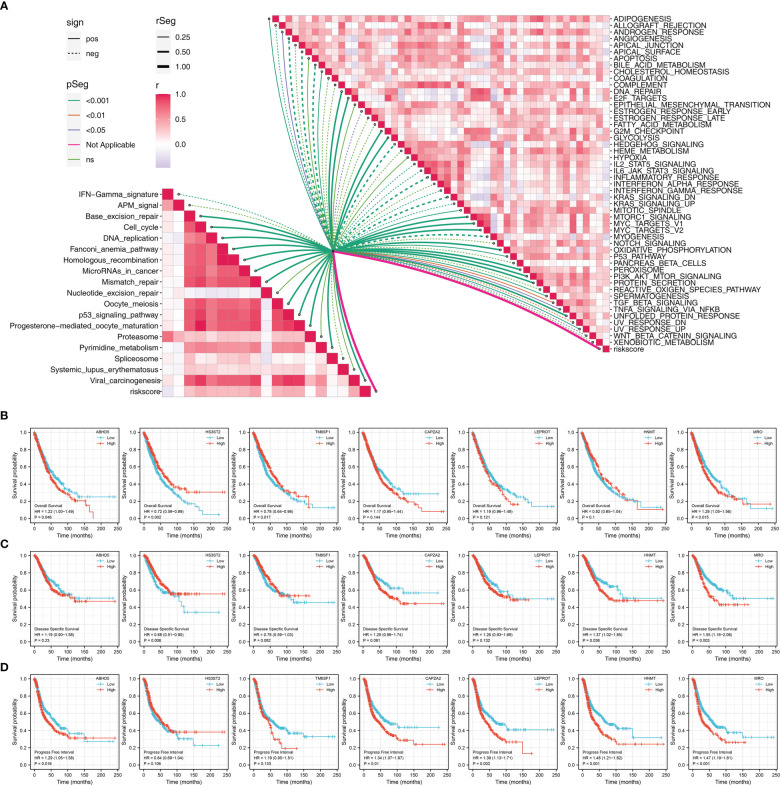
Pathway enrichment of the riskscore **(A)** Correlation of the riskscore and immune and metabolism pathways; **(B)** KM survival curves in OS of model genes; **(C)** KM survival curves in DSS of model genes; **(D)** KM survival curves in PFS of model genes.

**Figure 6:**
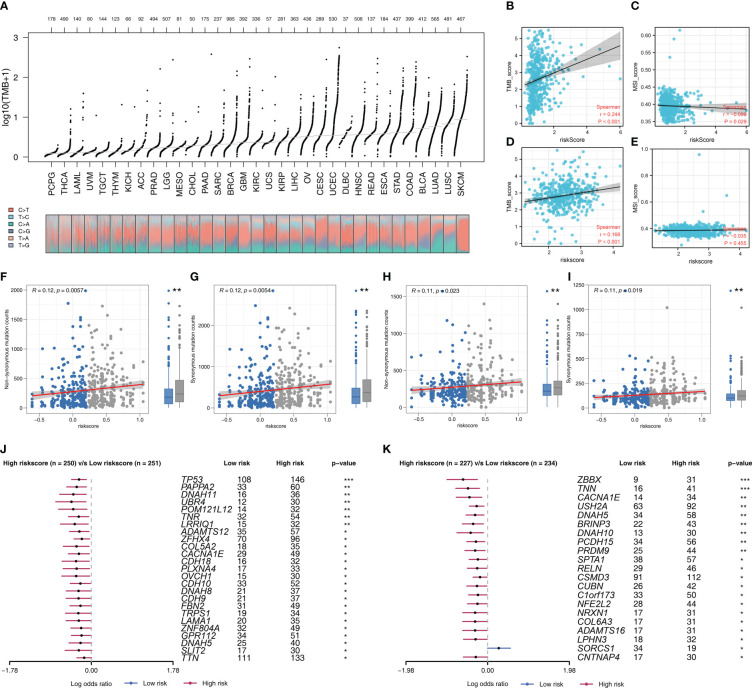
Genomic instability analysis **(A)** The overview of tumor mutation in pan-cancer; **(B, C)** The correlation of TMB and MSI score with riskscore in LUAD cohort; **(D, E)** The correlation of TMB and MSI score with riskscore in LUSC cohort; **(F, G)** The correlation of non-synonymous mutation counts and synonymous mutation counts with riskscore in LUAD cohort; **(H, I)** The correlation of non-synonymous mutation counts and synonymous mutation counts with riskscore in LUSC cohort; **(J)** The top mutated genes differentially existed in high and low risk LUAD patients; **(K)** The top mutated genes differentially existed in high and low risk LUSC patients.

Riskscore was positively correlated with most immunotherapy-related terms, including mismatch repair, cell cycle and DNA replication (**Figure 5A**). Additionally, we examined the correlation between riskscores and Hallmark gene pathway scores, from which a strong linear correlation can be observed between riskscore and multiple oncogenetic pathways, including G2M checkpoint, glycolysis, E2F targets, DNA repair, mTORC1 signaling and PI3K/AKT/mTOR signaling (**Figure 5A**). KM analysis was conducted on patients with different levels of the seven model genes in TCGA-LUDA, TCGA-LUSC and E-GEOD+GSE cohorts (**Figures 5B–D**). The results showed that ABHD5, CAPZA2, LEPROT and MRO might be the risk factor of NSCLC, while HS3ST2, TM6SF1, and HNMT might be the protective factors. In addition, as shown in **Figure 6A**, we identified a relatively big number of nonsynonymous somatic mutations in both LUAD patients and LUSC patients. Then TMB score and MSI score for each patient were calculated, and we found that riskscore was positively correlated with the TMB score in both LUAD patients and LUSC patients (**Figures 6B–E**). Somatic mutation data of LUAD patients and LUSC patients were also analyzed, and we found that a higher somatic mutation including non-synonymous, synonymous mutations was enriched in high risk patients (**Figures 6F–I**). After maftools analysis, differential mutated genes with p < 0.05 were identified. TP53, PAPPA2, DNAH11, UBR4, POM121L12, TNR, and LRRIQ1 mutated more often in high risk LUAD group (**Figure 6J**), while ZBBX, TNN, CACNA1E, USH2A, DNAH5, BRINP3, DNAH10, PCDH15 and PRDM9 mutated more often in high risk LUSC group (**Figure 6K**).

### Therapy prediction and potential drug identification

**Figure 7:**
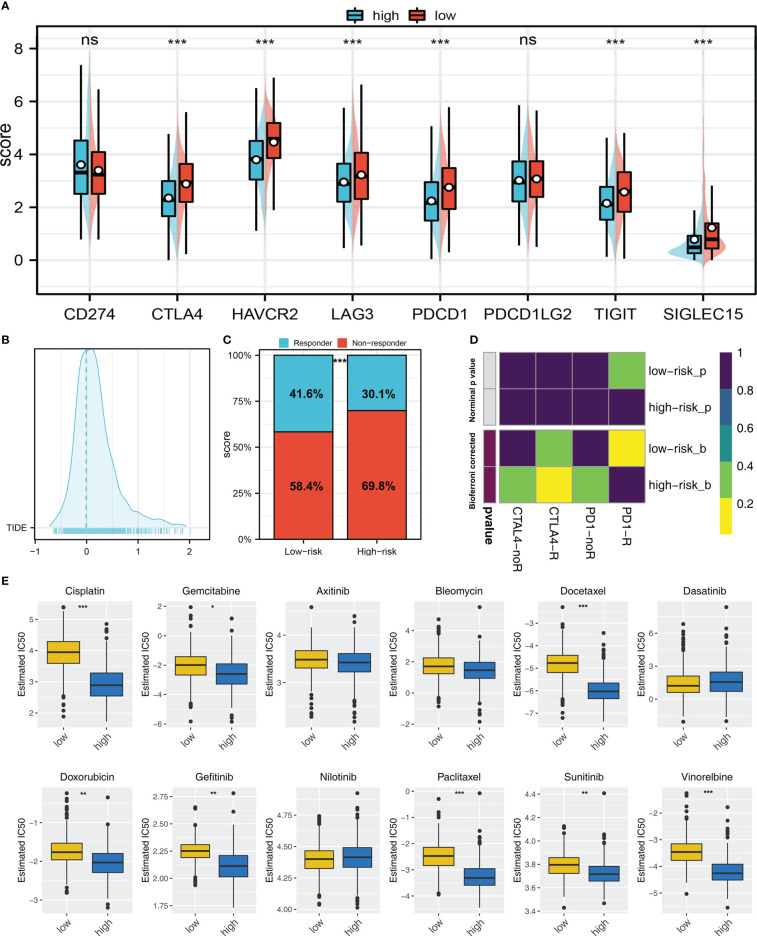
Immunotherapy and drug sensitivity **(A)** Important immune checkpoint expression in high and low risk patients; **(B)** The patients with TIDE score > 0 was regarded as non-responders and < 0 was regarded as responders; **(C)** A higher percentage of responders was observed in low risk group; **(D)** Submap algorithm showed that the low risk patients might be more sensitive to PD-1 therapy, while high risk patients might be more sensitive to CTLA-4 therapy; **(E)** The difference of chemotherapy sensitivity in high and low risk patients. ns = P > 0.05, * = P < 0.05, ** = P < 0.01, *** = P < 0.001.

Immune checkpoints exert an important role in cancer immunotherapy. Results indicated that patients with high and low risk exhibited significant differences in immune checkpoint expression, indicating the underlying difference of immunotherapy response rate (**Figure 7A**). Meanwhile, TIDE score was calculated to predict the likelihood of response to immunotherapy (**Figure 7B**). Results revealed patients with low riskscore may respond better to immunotherapy (**Figure 7C**). Besides, the subclass mapping algorithm was also applied to investigate the genomic similarity between patients in two risk groups and the patients that responded to immunotherapies. Interestingly, low risk patients are most likely to benefit from PD-1 therapy, while high risk patients may benefit from CTLA-4 therapy (**Figure 7D**). Furthermore, the GDSC database was employed in our analysis to estimate the IC50 of twelve commonly used drugs between two risk groups. Finally, the estimated IC50 of seven drugs differs significantly between two risk groups, including Cisplatin, Docetaxel, Doxorubicin, Gefitinib, Paclitaxel, Sunitinib and Vinorelbine (**Figure 7E**).

### HNMT enhances the proliferation ability of lung cancer

**Figure 8:**
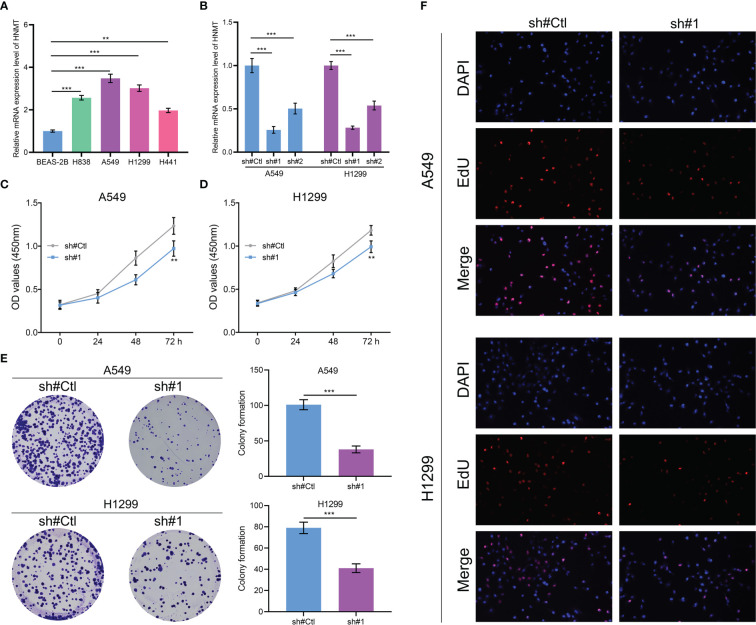
Role of HNMT in lung cancer **(A)** qRT-PCR was utilized to detect the RNA level of HNMT in lung cancer cells; **(B)** The knockdown efficiency of HNMT in lung cancer cells; **(C, D)** CCK8 assay was performed in control and HNMT knockdown cell; **(E)** Colony formation assay was performed in control and HNMT knockdown cell; **(F)** EdU assay was performed in control and HNMT knockdown cell. ** = P < 0.01, *** = P < 0.001.

HNMT was identified for further investigation. The qRT-PCR of cell lines indicated that HNMT was overexpressed in lung cancer cells (**Figure 8A**). A satisfactory knockdown efficiency was presented in **Figure 8B**. CCK8 and colony formation assay revealed that the knockdown of HNMT can remarkably weaken the cell proliferation ability of lung cancer cells (**Figures 8C–E**). Moreover, a lower number of EdU-positive cells was observed in the cell with HNMT knockdown (**Figure 8F**). These results indicated that the HNMT can promote lung cancer proliferation.

## Discussion

As of now, lung cancer continues to pose a major threat to global health. For most lung cancer patients at an early stage, surgery is the mainstay of treatment, and lobectomy is the preferred operation (24). However, it is noteworthy that most lung cancer patients are in an advanced stage when they receive their first diagnosis. Meanwhile, there remains controversy over the benefits of surgical therapy for lung cancer patients with advanced stage (25). Thus, exploration of the intrinsic mechanisms of NSCLC could help us identify novel diagnostic and therapeutic targets.

Macrophages could greatly affect cancer development and metastasis (26). The macrophage is both an antigen-presenting and immune cell. Macrophages are widely distributed and can specifically bind to tumors (27). In general, M2 macrophages play a cancer-promoting role in most malignancies, which could cause an immunosuppressive tumor microenvironment and are actively involved in cancer metastasis (28).

Here, we firstly explored the prognosis effect of M2 macrophages in NSCLC. Same with originally conceived, in multiple independent NSCLC cohorts, we noticed a poor prognosis in patients with high M2 macrophage infiltration. Next, we identified 114 M2 macrophage-related genes and established a prognosis model to predict patients OS based on seven genes, including ABHD5, HS3ST2, TM6SF1, CAPZA2, LEPROT, HNMT and MRO. KM survival curves and ROC survival curves revealed that our signature was reliable. Furthermore, results of biological enrichment showed that the pathway of DNA repair, G2M checkpoint, E2F targets, glycolysis, mTORC1 signaling and PI3K/AKT/mTOR signaling were aberrantly activated in the high risk group. Signaling pathways PI3K/AKT/mTOR, which is a classic pathway with a wide investigation, play a vital role in the proliferation and differentiation of cells (29). The G2/M checkpoint is the second checkpoint of the cell cycle and its abnormality of it might result in cycle disturbance (30). One of the main causes of cancer outbreaks is the change in DNA repair pathways. Meanwhile, compared with normal cells, tumor cells are more susceptible to DNA damage (31). These results showed that the aberrant activation of these oncogenic pathways might may result in a worse prognosis.

Our prognosis based on the ABHD5, HS3ST2, TM6SF1, CAPZA2, LEPROT, HNMT and MRO showed great prediction efficiency on patients OS. Meanwhile, these seven model genes were associated with higher M2 macrophage infiltration in NSCLC tissue. Liang and their colleagues found that cancer-derived exosomal TRIM59 could physically bind with ABHD5, further regulating macrophage and lung cancer progression (32). Hwang and their colleagues indicated that HS3ST2 had a high methylation signature in NSCLC cells, which could significantly lung cancer development (33). Zhong and their colleagues revealed that the TM6SF1 was related to the NSCLC tumor Microenvironment (34). Kuo and their colleagues found that the upregulation of HNMT could induce tumor stemness in NSCLC (35). MRO and CAPZA2 have not been reported in NSCLC. Our result showed that these genes are associated with M2 macrophages and might be the potential biomarker of NSCLC.

Immunotherapy, including PD-1/PD-L1 checkpoint blockade immunotherapy, has initiated a novel era of cancer treatment. Recently, a new computational method referred to as TIDE has been developed to model tumor immune evasion, demonstrating strong clinical utility for immunotherapy research (36). In our research, we first performed differential expression analysis on multiple important immune checkpoints (SIGLEC15, TIGIT, HAVCR2, PDCD1, CTLA4, LAG3, and PDCD1LG2) between two risk groups, and we found these immune checkpoints were expressed ubiquitously with high expression in high risk groups. In addition, TIDE analysis revealed that patients who responded to immunotherapy accounted for more in the low risk group. Collectively, the subclass mapping algorithm was developed to evaluate similarities of expression matrix in responding to immunotherapies (37), showing the same results that immunotherapy was more effective in patients with low riskscore. All results suggested that immunotherapy efficacy could be predicted by our model. Moreover, the GDSC database was employed in our study, and we found the estimated IC50 of seven drugs differs significantly between two risk groups, including Cisplatin, Docetaxel, Doxorubicin, Gefitinib, Paclitaxel, Sunitinib and Vinorelbine, aiding clinicians in helping tailor therapy accordingly.

On the whole, our study identified the molecules significantly affecting M2 macrophage infiltration and identified a prognosis signature that robustly indicated patients prognosis. Moreover, we validated the cancer-promoting effect of HNMT using *in vitro* experiments. However, there are still several limitations that should be noted. Firstly, though a comprehensive search for public databases including appropriate expression matrix and clinical information was performed, further validation of our findings should be conducted in other cohorts. Secondly, M2 macrophages and genes related to them need to be studied further.

## Data availability statement

The datasets presented in this study can be found in online repositories. The names of the repository/repositories and accession number(s) can be found in the article/**Supplementary Material**.

## Author contributions

ZZ, YJ and YZ contributed equally to this work. ZZ, ZY and KD conducted all the analysis. ZZ and JY wrote the manuscript. YC and LZ designed this work. All authors contributed to the article and approved the submitted version.

## Supplementary material

Supplementary Figure 1 GO analysis of M2 macrophages in lung cancer patients **(A)** GO analysis of M2 macrophage in LUAD patients; **(B)** GO analysis of M2 macrophage in LUSC patients.

Supplementary Figure 2 KEGG analysis of M2 macrophages in lung cancer patients **(A)** KEGG analysis of M2 macrophage in LUAD patients; **(B)** KEGG analysis of M2 macrophage in LUSC patients.
