# Supplementary figures and images for: Investigation of M2 macrophage-related gene affecting patients prognosis and drug sensitivity in non-small cell lung cancer: Evidence from bioinformatic and experiments
Source: Front Oncol. 2022 Dec 15;12:1096449. doi: 10.3389/fonc.2022.1096449 (PMC9797692; doi:10.3389/fonc.2022.1096449)

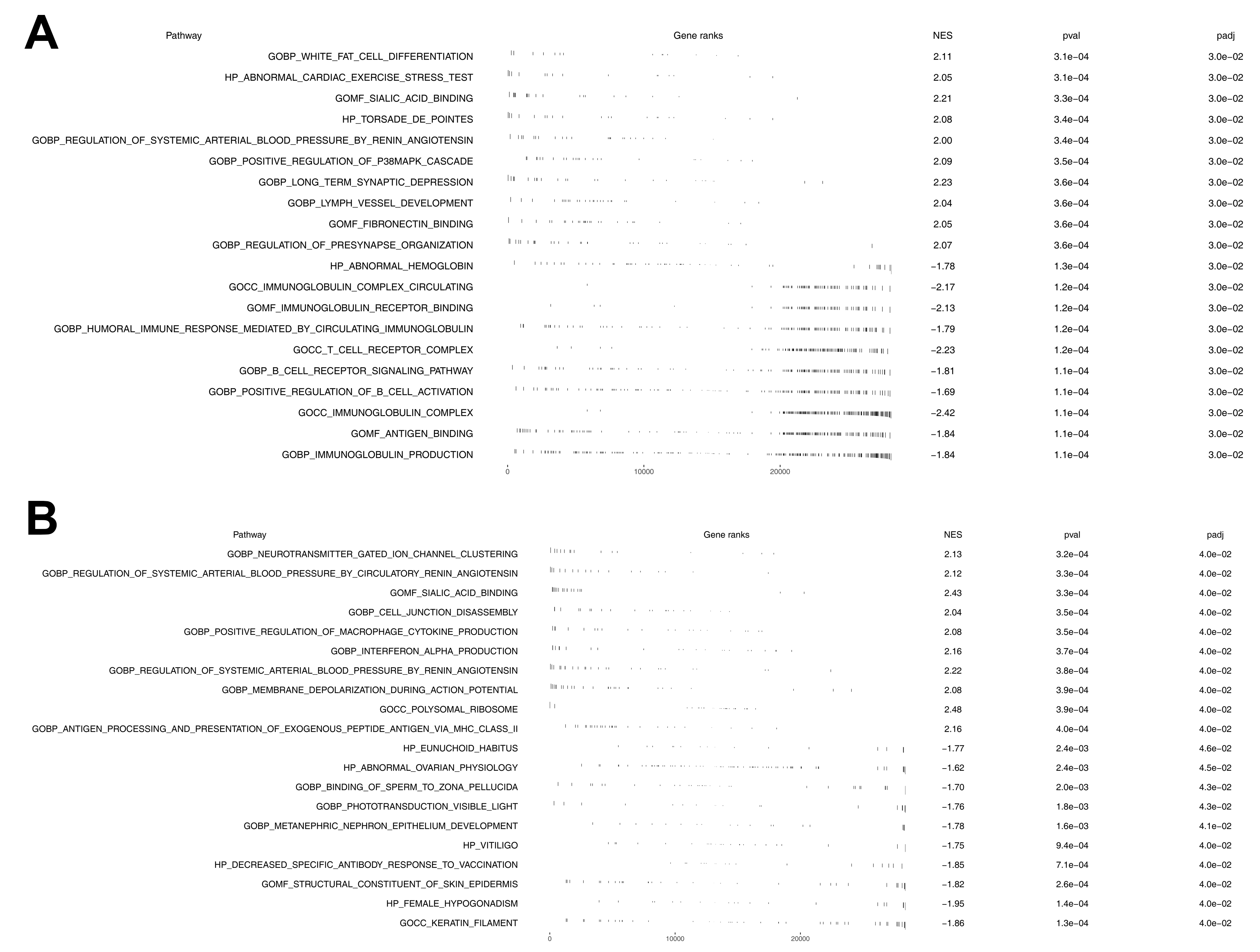

Supplement: Supplementary Figure 1 — GO analysis of M2 macrophages in lung cancer patients (A) GO analysis of M2 macrophage in LUAD patients; (B) GO analysis of M2 macrophage in LUSC patients. [file Image_1.tif]

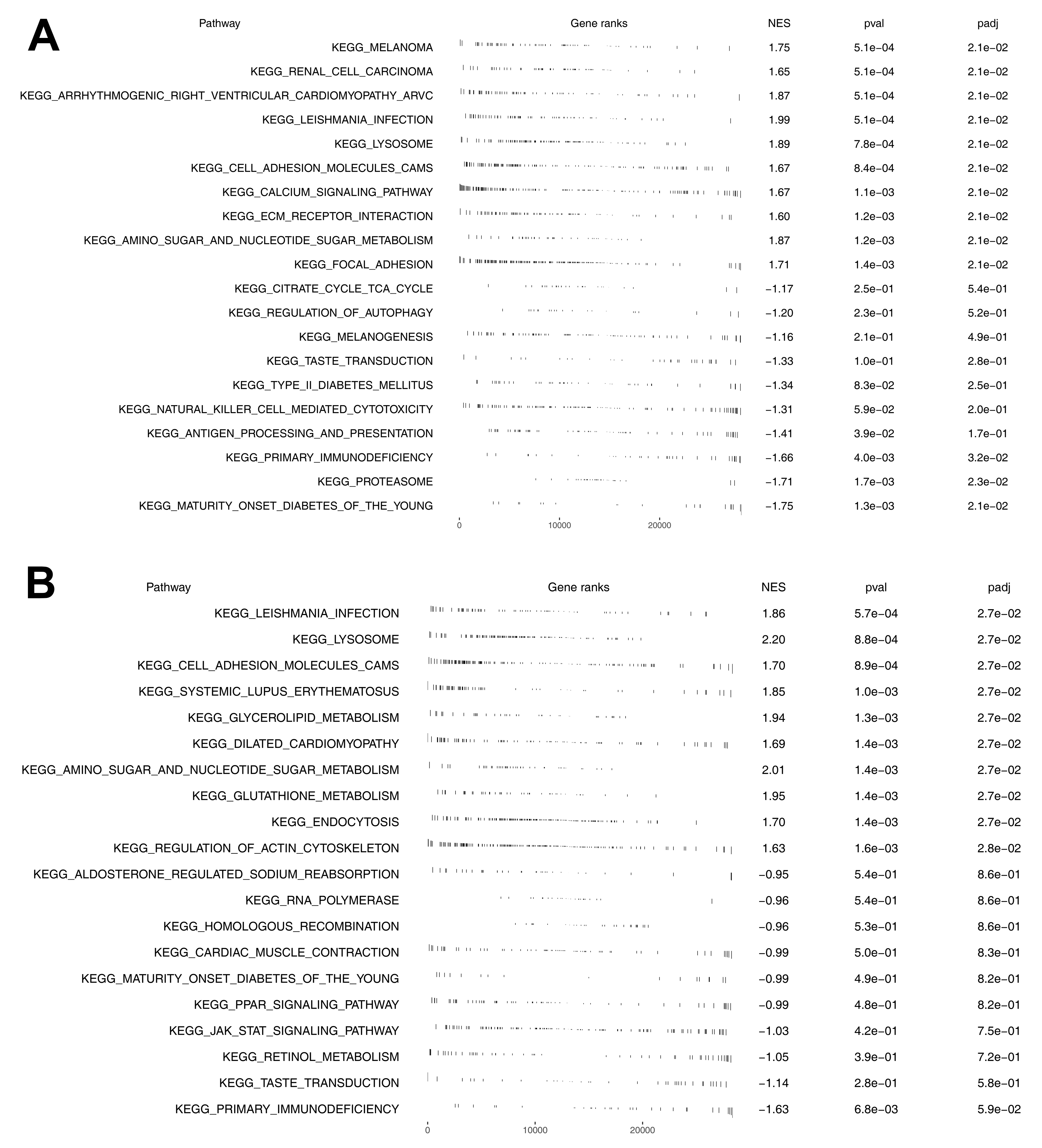

Supplement: Supplementary Figure 2 — KEGG analysis of M2 macrophages in lung cancer patients (A) KEGG analysis of M2 macrophage in LUAD patients; (B) KEGG analysis of M2 macrophage in LUSC patients. [file Image_2.tif]
